# Impact of modification to DSM-5 criterion A for hypomania/mania in newly diagnosed bipolar patients: findings from the prospective BIO study

**DOI:** 10.1186/s40345-020-00219-9

**Published:** 2021-05-03

**Authors:** Mette U. Fredskild, Sharleny Stanislaus, Klara Coello, Sigurd A. Melbye, Hanne Lie Kjærstad, Kimie Stefanie Ormstrup Sletved, Trisha Suppes, Maj Vinberg, Lars Vedel Kessing

**Affiliations:** 1 Copenhagen Affective Disorders Research Centre (CADIC), Psychiatric Centre Copenhagen, Department O, 6233, Rigshospitalet, Blegdamsvej 9, 2100 Copenhagen, Denmark; 2 Department of Psychiatry & Behavioral Sciences, Stanford University School of Medicine, Palo Alto, CA USA; 3 Department of Clinical Medicine, Faculty of Health and Medical Sciences, University of Copenhagen, Copenhagen, Denmark; 4 Psychiatric Research Unit, Psychiatric Centre North Zealand, Hillerød, Denmark

**Keywords:** Bipolar disorder, Diagnostic and Statistical Manual of Mental Disorders Version IV (DSM-IV), Diagnostic and Statistical Manual of Mental Disorders 5th edition (DSM-5), Mood, Irritability, Energy, Activity, The International Classification of Diseases 10 (ICD-10), The International Classification of Diseases 11 (ICD-11)

## Abstract

**Background:**

DSM-IV states that criterion A for diagnosing hypomania/mania is mood change. The revised DSM-5 now states that increased energy or activity must be present alongside mood changes to diagnose hypomania/mania, thus raising energy/activity to criterion A. We set out to investigate how the change in criterion A affects the diagnosis of hypomanic/manic visits in patients with a newly diagnosed bipolar disorder.

**Results:**

In this prospective cohort study, 373 patients were included (median age = 32; IQR, 27–40). Women constituted 66% (n = 245) of the cohort and 68% of the cohort (n = 253) met criteria for bipolar type II, the remaining patients were diagnosed bipolar type I. Median number of contributed visits was 2 per subject (IQR, 1–3) and median follow-up time was 3 years (IQR, 2–4). During follow-up, 127 patients had at least one visit with fulfilled DSM-IV criterion A. Applying DSM-5 criterion A reduced the number of patients experiencing a hypomanic/manic visit by 62% at baseline and by 50% during longitudinal follow-up, compared with DSM-IV criterion A. Fulfilling DSM-5 criterion A during follow-up was associated with higher modified young mania rating scale score (OR = 1.51, CL [1.34, 1.71], p < 0.0001) and increased number of visits contributed (OR = 1.86, CL [1.52, 2.29], p < 0.0001).

**Conclusion:**

Applying the stricter DSM-5 criterion A in a cohort of newly diagnosed bipolar patients reduced the number of patients experiencing a hypomanic/manic visit substantially, and was associated with higher overall young mania rating scale scores, compared with DSM-IV criterion A. Consequently, fewer hypomanic/manic visits may be detected in newly diagnosed bipolar patients with applied DSM-5 criterion A, and the upcoming ICD-11, which may possibly result in longer diagnostic delay of BD as compared with the DSM-IV.

## Background

The introduction of the Diagnostic and Statistical Manual of Mental Disorders 5th edition (DSM-5) (Association AP 2013) has in many ways changed the diagnostic criteria for bipolar disorder (BD). Several iterations of the DSM, including DSM-IV, emphasized mood abnormalities (*elevated, expansive or irritable mood*) as the cardinal symptom (criterion A) for a hypomanic/manic episode, and listed psychomotor disturbances (activity) as one of the seven criterion B symptoms (Association AP 1994). However, the revised DSM-5, which was published in 2013, now states that increased energy or activity must be present alongside mood disturbances to diagnose a hypomanic/manic episode, thus raising psychomotor disturbances to criterion A (Association AP 2013) (see Table 1). The argument behind the modification of criterion A was that it might improve specificity when making the diagnosis of hypomania/mania, and consequently, BD.

**Table 1:** DSM and study definitions

	DSM definition of criterion A for a hypomanic/manic episode	Study defined criterion A for a hypomanic/manic visit based on YMRS^a^
DSM-IV	*“A distinct period of abnormally and persistently elevated, expansive or irritable mood”*	Item 1^b^ $\ge$ 2 and/or Item 5^d^ $\ge$ 4
DSM-5	*“A distinct period of abnormally and persistently elevated, expansive or irritable mood AND persistently increased activity or energy”*	Item 1 $\ge$ 2 and/or Item 5 $\ge$ 4 ***AND*** Item 2^c^ $\ge$ 2

^a^Young Mania Rating Scale (11-item)

^b^Item 1: Mood elevation

^c^Item 2: Energy/activity

^d^Item 5: Irritability

The International Classification of Diseases (ICD) is following in the footsteps of the DSM definitions. The ICD-10 definition of hypomania (F30.0)/mania (F30.1) highlighted mood abnormalities as criterion A (like DSM-IV) in the diagnostic criteria for research version (WHO 1992a) while the clinical descriptions and diagnostic guidelines version does not operate with A and B criteria (WHO 1992b). However, the newly online released ICD-11, like DSM-5, includes increased energy or activity as criterion A alongside mood abnormalities (WHO 2020).

Few studies have investigated the impact of the modification to DSM-5 criterion A for the diagnosis of hypomanic/manic episodes but only in cohorts of more chronic patients with more progressed and longer duration recurrent bipolar disorder. The STEP-BD study (Machado-Vieira et al. 2017), from the US, found that applying DSM-5 criterion A reduced the identified number of hypomanic/manic episodes by 48% when looking at baseline visits only (point prevalence), compared with DSM-IV criterion A. A study by our group of 907 chronic bipolar patients, from the US and Europe, found a 34% reduction in the number of patients experiencing a hypomanic/manic visit during follow up when DSM-5 criterion A was applied (Fredskild et al. 2019). On the other hand, the BDRN study (Gordon-Smith et al. 2017), from the UK, found that up to 94% of patients with a *lifetime* diagnosis of DSM-IV BD also met lifetime DSM-5 criteria for BD. All three mentioned studies have in common that they investigated populations of more chronic bipolar patients with more progressed and longer duration recurrent bipolar disorder. Results from the three mentioned studies suggest that the reduction in the diagnostic prevalence of hypomania/mania, and consequently BD, is highest earlier in the course of bipolar illness (a 34–48% reduction) but may diminish during lifetime (to a 6% reduction).

Thus, a knowledge gap remains, regarding how the introduction of DSM-5 criteria for BD affects the diagnostic prevalence of hypomanic/manic visits in newly diagnosed bipolar patients and potentially prolong the diagnostic gap from first symptoms to diagnosis.

Therefore, in a cohort of newly diagnosed patients with BD, we aimed to quantitatively determine the point prevalence of patients fulfilling criterion A of a hypomanic/manic visit according to DSM-IV and DSM-5 at baseline and during longitudinal follow up. Furthermore, we aimed to investigate if any of the covariates available could predict DSM-5 diagnosis. Lastly, we explored the individual association between manic symptoms available from the Young Mania Rating Scale (YMRS) to understand the relationship between present manic symptoms and the transition from DSM-IV to DSM-5 diagnosis of hypomania/mania.

We hypothesized that applying DSM-5 criterion A would lower the number of hypomanic/manic visits significantly, both at baseline and during follow-up and that no association between sex, age and DSM-5 diagnosis would be found. Lastly, we hypothesized that mood abnormalities (mood elevation and irritability) and energy/activity would show a strong association.

This study is of high importance as the consequences of modification to DSM-5 criterion A are still unknown and have never been looked at in a population of newly diagnosed bipolar patients. This study will help us understand the impact of the current modification to DSM-5 criterion A for newly diagnosed bipolar patients, and hopefully help us understand the patient’s disease-trajectory in the light of DSM-5.

## Methods

### Study design and aim

The present study is a prospective cohort study investigating follow-up data on newly diagnosed patients with BD. The overall aim of the present study is to elucidate the clinical consequences of the modification to the current DSM-5 criterion A when focusing on newly diagnosed bipolar patients.

### Setting of the study

The present study investigated data from the ongoing longitudinal Bipolar Illness Onset study (BIO), which aims to identify biomarkers for BD (Kessing et al. 2017). Details on methods and procedures in the BIO-study are fully described elsewhere (Kessing et al. 2017). The BIO-study protocol was approved by the Committee on Health Research Ethics of the Capital region of Denmark (Protocol No. H-7-2014-007) and the Danish Data Protection Agency, Capital Region of Copenhagen (RHP-2015-023). All participants provided written informed consent. The BIO-study complied with the Declaration of Helsinki principles (Seoul, October 2008).

### Participants

We included 373 patients with newly diagnosed BD. Recruitment of participants took place at the outpatient Copenhagen Affective Disorder Clinic from June 2015 to November 2019. The Clinic provides treatment for patients with newly diagnosed BD and receives patients from the Capital region of Denmark, an area covering 1.6 million and all the psychiatric centers in the Region (Kessing et al. 2013). All patients referred to the clinic as newly diagnosed bipolar patients were routinely asked for inclusion in the BIO-study.

At baseline, the initial diagnostic assessment of participants was conducted by an experienced specialist in psychiatry using the Structures Clinical Interview for DSM-IV-TR Axis I Disorders (First et al. 1996), categorizing patients into bipolar disorder I (BD I) or bipolar disorder II (BD II). The diagnosis of a single manic episode or BD was confirmed in a semi-structured research-based interview by M.Ds or M.Scs in Psychology Ph.D. students using the Schedules for Clinical Assessment in Neuropsychiatry (SCAN) providing an ICD-10 diagnosis (WHO 1992b). Inclusion criteria were an ICD-10 diagnosis of BD or a single manic episode and age 15–70 years.

No attempts to balance the prevalence of bipolar subtypes were taken. Patients with BD not otherwise specified and patients with cyclothymia were not offered treatment in the Clinic and thus not included in the BIO-study. Exclusion criteria were organic BD secondary to brain injury.

Patients with BD received treatment as usual in the clinic without interference from study investigators.

Besides the clinical assessment at inclusion (baseline), patients were assessed during remitted depressive, manic or mixed phases and and once yearly for up to 5 years of follow up.

### Procedures

At each assessment the severity of manic and/or depressive symptoms were evaluated using the 11-item clinician administered Young Mania Rating Scale (YMRS) (Young et al. 1978) and the 17-item Hamilton Depression Rating Scale (HAMD-17) (Hamilton 1960), respectively, covering the precceding 3 days. Both rating scales have been extensively validated for assessing manic and depressive symptoms, respectively (Young et al. 1978; Bech 2012). Patients were required to have a completed YMRS score at their first visit (baseline) to be included in the present study, as this was the basis of inclusion criteria.

### Definitions

We used the same methodology and definitions as in our prior publication on patients with more chronic BD (). The individual ratings on item 1 (Mood), item 2 (Energy/Activity) and item 5 (Irritability) on the 11-item YMRS allowed an evaluation of the presence of fulfilled criterion A of hypomania/mania according to DSM definition (see Table 1).

A score equal or above 2 on item 1 (Mood) was considered reflective of increased/elevated mood. A score equal or above 2 on item 2 (Energy/Activity) was considered reflective of increased motor activity or energy. A score equal or above 4 on item 5 (Irritability) was considered reflective of increased irritability (see Table 1).

Thus, study definition of fulfilling DSM-IV criterion A for a hypomanic/manic visit was defined as item 1 (Mood) $\ge$ 2 and/or item 5 (Irritability) $\ge$ 4, presumably adequate to meet the DSM-IV definition of criterion A. Study definition of fulfilled DSM-5 criterion A for a hypomanic/manic visit was defined as item 1 (Mood) $\ge$ 2 and/or item 5 (Irritability) $\ge$ 4 AND item 2 $\ge$ 2 (Energy/Activity), presumably adequate to meet the DSM-5 definition of criterion A (see Table 1).

Depressive symptoms were defined as an HAMD-17 total score $\ge$ 15.

### Statistical analyses

A total of 849 visits by 373 participants were analyzed. Data analyses were performed based on participants (373 patients) using logistic regression models and based on visits (849 visits) using linear mixed-effects models.

Descriptive data were presented as median and interquartile range (IQR) or count and percentage (%). A power of 80% and a significance level of alfa = 0.05 were chosen for all statistical analyses. Results from analyses were presented as odds ratios with 95% confidence limits.

All statistical analyses were performed using software programs (SAS Enterprise Guide version 7.15, SAS institute, Cary, North Carolina).

### Missing data

One subject had missing data on the variables “age of onset” and “age at first hypomanic/manic episode” and additional one subject had missing data on “delay in diagnosis”. These missing data were handled by single imputation with the median value of the cohort. Twelve subjects had missing data on family history of BD, 4 had missing data on no. of previous hospitalizations/admissions, and 4 had missing data on education years. These missing data were likewise handled by imputation before inclusion in the logistic regression model. A maximum of 7 missing values on the individual YMRS items were present throughout all visits (849) and were handled in the mixed model analysis.

### Logistic regression

A modified YMRS score for each visit was calculated based on the total YMRS score, minus item 1, 2, and 5, as these items were used to define the outcome, i.e., DSM-IV and DSM-5 criterion A. The mean modified YMRS score was found for each participant as a mean of the modified YMRS scores across their visits.

Logistic regression models were performed in order to investigate if any of independent variables could predict DSM-5 Criterion A fulfillment during follow-up. The outcome was defined as fulfilling DSM-5 criterion A at least once during follow-up in study (yes/no). Univariate and multivariable analyses were performed, and data were represented unadjusted and fully adjusted for bipolar subtype, sex, no. of visits contributed, age first hypomanic/manic episode (years), no. of visits with depressed symptoms, mean modified YMRS total score, family history of BD, delay in diagnosis, education years. BD subtype, family history of BD, and sex were treated as binary variables, whereas the remaining variables were treated as continuous variables in the univariate and multivariable analysis. Variables included in the multivariable analysis were included based on clinical relevance and/or significance in univariate analysis (p < 0.2). Bonferroni correction was conducted to correct for multiple testing.

### Mixed models

Based on visits, we examined the association between individual items on the YMRS. Visits data (849 visits) represent repeated measures data and were analyzed accordingly, using generalized linear mixed-effects models, specifying each participant as random effect. The data represented zero-inflated data. Thus, the outcome was dichotomized, and the mixed models were specified as binomial.

The first analysis investigated the individual YMRS items (predictors) and their association with experiencing increased mood (outcome variable). The outcome variable mood (item 1) was defined as elevated (> 0) and not elevated (= 0). Univariate as well as multivariable linear mixed-effects models were conducted, and results presented unadjusted and fully adjusted for the other YMRS items. The analysis was repeated with irritability (item 5) as the outcome.

The analyses were specified with mood and irritability, respectively, as outcome variable, the individual YMRS items as predictors, and a random intercept for each participant to account for multiple observations made on the same subject.

## Results

### Demographic and clinical characteristics

We included 373 patients (median age = 32; IQR 27–40; 66% were female (n = 245) and 32% (n = 120) met criteria for BD I. A total of 849 visits by the 373 patients were included.

The median age at onset of BD was 18 (IQR, 15–22). Median number of visits contributed by each subject in the study was 2 (IQR, 1–3), corresponding to a median follow-up time in months by each subject of 41 months (IQR, 26–50) (see Table 2).

**Table 2:** Sociodemographic and clinical characteristics

Patients, n	373
Total visits	849
Sex	
Male, n (%)	128 (34%)
Female, n (%)	245 (66%)
Bipolar subtype	
BD I, n (%)	120 (32%)
BD II, n (%)	253 (68%)
Years of education, median (IQR)	15 (13–17)
No. of previous hospitalizations/admissions, median (IQR)	0 (0–1)
Family history of BD^a^, n (%)	56 (16%)
Age (years), median (IQR)	32 (27–40)
Age at onset (years)^b^, median (IQR)	18 (15–22)
Age at first hypomanic/manic episode (years)^c^, median (IQR)	21 (17–27)
Illness duration (years)^d^, median (IQR)	10 (6–16)
Delay in diagnosis (years)^e^, median (IQR)	5 (2–11)
Follow up time in study (months), median (IQR)	41 (26–50)
Follow up time in study (years), median (IQR)	3 (2–4)
No. of visits contributed per patient, median (IQR)	2 (1–3)
YMRS total score, median (IQR)	3 (0–6)
Modified YMRS total score^f^, median (IQR)	1 (0–4)
No. of patients fulfilling a depressed visit through follow up (Hamilton ≥ 15), n (%)	135 (36%)
No. of patients fulfilling a visit with DSM-IV criterion A for hypomania/mania through follow up, n (%)	127 (34%)
No. of patients fulfilling a visit with DSM-5 criterion A for hypomania/mania through follow up, n (%)	63 (17%)
**Baseline visits (first visit by each subject), n**	373
YMRS total score at first visit, median (IQR)	3 (0–6)
Modified YMRS total score at first visit, median (IQR)	1 (0–4)
No. of patients fulfilling DSM-IV criterion A for hypomania/mania at baseline, n (%)	86 (23%)
No. of patients fulfilling DSM-5 criterion A for hypomania/mania at baseline, n (%)	33 (9%)
YMRS total score for participants fulfilling DSM-IV criterion A at first visits, median (IQR)	10 (7–14)
Modified YMRS total score for participants fulfilling DSM-IV criterion A at first visits, median (IQR)	5 (2–8)
YMRS total score for participants fulfilling DSM-5 criterion A at first visits, median (IQR)	14 (10–16)
Modified YMRS total score for participants fulfilling DSM-5 criterion A at first visits, median (IQR)	8 (4–10)
No. of patients with a depressive episode at first visit (Hamilton ≥ 15) at baseline, n (%)	101 (27%)

^a^Family history of BD: Mother, father or sibling with bipolar disorder

^b^Age at onset: From birthdate to first episode of depression, mania, hypomania or mixed episode

^c^Age at first hypomanic/manic episode: From birthdate to first mania, hypomania or mixed episode

^d^Illness duration: From first episode (depression, mania, hypomania or mixed episode) to date of inclusion

^e^Delay in diagnosis: From the time where the first mania, hypomania or mixed episode occurred to date of the diagnosis of a single manic or mixed episode or a diagnosis of BD

^f^Modified YMRS total score = total score minus item 1, item 2 and item 5

### DSM-IV and DSM-5 criterion A

#### Baseline/first visit

At baseline (the participant’s first visit), 86 patients fulfilled criterion A of a hypomanic/manic visit according to DSM-IV criterion A (mood and/or irritability). Of the 86 patients, 33 patients fulfilled DSM-5 criterion A (mood and/or irritability AND energy/activity) of a hypomanic/manic visit, a reduction of 62% at baseline (see Table 2).

The median of the modified YMRS total score at baseline was 5 (IQR, 2–8) in the group of 86 patients who fulfilled DSM-IV criterion A and 8 (IQR, 4–10) in the group of 33 patients who fulfilled DSM-5 criterion A (see Table 2).

#### Follow up

During follow-up, 127 subjects had at least one visit fulfilling DSM-IV criterion A of a hypomanic/manic visit. Applying DSM-5 criterion A, 63 subjects had at least one visit fulfilling DSM-5 criterion A of hypomania/mania corresponding to a reduction of 50% in the number of subjects who experienced a hypomanic/manic visit according to criterion A during follow-up in the study when applying DSM-5 criterion A (see Table 2).

### Multivariable logistic regression

We investigated if any of the demographic and clinical variables could predict DSM-5 diagnosis of hypomania/mania according to DSM-5 criterion A during follow-up. The multivariable logistic regression model revealed that the number of visits contributed and the mean modified YMRS total were associated with DSM-5 diagnosis according to criterion A during follow-up (see Table 3). For every 1 visit contributed, the odds of DSM-5 diagnosis according to criterion A, increased by 86% after adjustment (adjusted OR [95% CL] 1.86 [1.52, 2.29], p > 0.0001). For every 1 increasement in mean modified YMRS total score, the odds of DSM-5 diagnosis increased by 51% after adjustment (adjusted OR [95% CL] 1.51 [1.34, 1.71], p > 0.0001).

**Table 3:** Logistic regression—prediction of DSM-5 criterion A fulfillment in 373 subjects

Variable	OR	95% CL	p-value
Age (years)			
Univariate analysis	0.99	0.97, 1.02	0.65
Sex (Male vs female)			
Univariate analysis	0.95	0.53, 1.68	0.86
Multivariate analysis^f^	0.79	0.37, 1.67	0.53
No. of visits contributed			
Univariate analysis	1.51	1.31, 1.75	< 0.0001
Multivariate analysis^f^	1.86	1.52, 2.29	< 0.0001**
Bipolar subtype (BD I vs BD II)			
Univariate analysis	1.50	0.85, 2.61	0.16
Multivariate analysis^f^	1.64	0.82, 3.27	0.16
Age at onset (years)^a^			
Univariate analysis	0.97	0.92, 1.01	0.11
Age first hypomanic/manic episode (years)^b^			
Univariate analysis	0.96	0.92, 0.99	0.03
Multivariate analysis^f^	0.99	0.94, 1.04	0.57
Delay in diagnosis (years)^c^			
Univariate analysis	1.02	0.99, 1.06	0.15
Multivariate analysis^f^	1.03	0.98, 1.07	0.25
No. of visits with depressed symptoms			
Univariate analysis	1.34	0.99, 1.81	0.06
Multivariate analysis^f^	0.78	0.51, 1.19	0.24
Mean modified YMRS total score^d^			
Univariate analysis	1.38	1.24, 1.52	< 0.0001
Multivariate analysis^f^	1.51	1.34, 1.71	< 0.0001**
Number of previous hospitalizations/ admissions			
Univariate analysis	0.92	0.74, 1.14	0.44
Education years			
Univariate analysis	0.92	0.83, 1,02	0.12
Multivariate analysis^f^	0.90	0.78, 1.03	0.13
Family history of BD^e^ (no vs yes)			
Univariate analysis	0.79	0.35, 1.77	0.57
Multivariate analysis^f^	0.71	0.26, 1.97	0.52

^a^Age at onset: From birthdate to first episode of depression, mania, hypomania or mixed episode

^b^Age at first hypomanic/manic episode: From birthdate to first mania, hypomania or mixed episode

^c^Delay in diagnosis: From first mania, hypomania or mixed episode to date of BD diagnosed

^d^Mean of the modified YMRS total score (YMRS total score minus item 1, item 2 and item 5) across patient visits

^e^Family history of BD: Mother, father or sibling with bipolar disorder

^f^Adjusted for bipolar subtype, sex, no. of visits contributed, age first hypomanic/manic episode (years), no. of visits with depressed symptoms, mean modified YMRS total score, family history of BD, delay in diagnosis, education years

^**^Remained statistically significant after Bonferroni correction for multiple testing in the multivariate analysis (p < 0.006)

Age at first hypomanic/manic episode was associated with fulfilling DSM-5 diagnosis according to criterion A in univariate analysis (OR = 0.96, 95% CL [0.92, 0.99], p = 0.03), but after adjustment in the multivariable analysis, this association was no longer statistically significant (OR = 0.99, 95% CL [0.94, 1.04], p = 0.57).

Delay in diagnosis, no. of visits with depressed symptoms, and education years all showed a borderline statiscal significant association with DSM-5 diagnosis according to criterion A in univariate analysis but these associations were no longer statistically significant after adjustment in the multivariable analysis (see Table 3).

We did not find any statistically significant associations between sex, age, bipolar subtype, no. of previous hospitalizations/admissions, family history of BD and DSM-5 diagnosis according to criterion A.

Sensitivity analyses were performed, stratifying based on bipolar subtype and sex, respectively, however, this did not change the results.

### Association between individual YMRS items

#### Mood

We investigated the individual hypomanic/manic symptoms (individual YMRS items) and their relationship with mood elevation (see Table 4). In the univariate analysis all items except item 11 (Insight) showed association with mood elevation before adjustment. After adjustment and Bonferroni correction, only item 2 (Energy/Activity), item 3 (Sexual Interest), item 4 (Sleep), and item 6 (Speech), remained statistically significantly associated with item 1 (Mood) with a p-value < 0.005. For every 1-value increasement in Energy/Activity on item 2, the odds of experiencing elevated mood increased by 2.5 times in the adjusted model (adjusted OR [95% CL] 2.5 [1.8, 3.4]) (see Table 4).

**Table 4:** Odds ratios of experiencing elevated mood depending on the different manic symptoms (YMRS items) during 849 visits

		OR	95% confidence limts	Range of scale in data set^a^
Item 1 (Mood)	x	x	X	0–4
Item 2 (Energy/activity)	Unadjusted	4.9	(3.8, 6.2)	0–4
	Adjusted^b^	2.5*	(1.8, 3.4)*	
Item 3 (Sexual interest)	Unadjusted	4.3	(3.3, 5.6)	0–3
	Adjusted	2.2*	(1.6, 3.2)*	
Item 4 (Sleep)	Unadjusted	2.7	(2.2, 3.4)	0–4
	Adjusted	1.6*	(1.2, 2.1)*	
Item 5 (Irritability)	Unadjusted	1.4	(1.2, 1.5)	0–6
	Adjusted	0.9	(0.7, 1.1)	
Item 6 (Speech)	Unadjusted	2.9	(2.4, 3.4)	0–6
	Adjusted	2.0*	(1.6, 2.4)*	
Item 7 (Language-Thought Disorder)	Unadjusted	2.2	(1.9, 2.7)	0–4
	Adjusted	1.3	(1.0, 1.7)	
Item 8 (Content)	Unadjusted	2.2	(1.8, 2.7)	0–8
	Adjusted	1.2	(0.9, 1.6)	
Item 9 (Aggression)	Unadjusted	1.7	(1.1, 2.4)	0–5
	Adjusted	0.8	(0.5, 1.6)	
Item 10 (Appearance)	Unadjusted	2.4	(1.4, 4.2)	0–4
	Adjusted	0.5	(0.2, 1.3)	
Item 11 (Insight)	Unadjusted	1.5	(0.9, 2.4)	0–6
	Adjusted	0.7	(0.3, 1.5)	

Outcome Mood > 0 vs mood = 0

^a^Range of scale: observed minimum and maximum values of the individual YMRS items in data set

^b^Adjusted model is adjusted for all the remaining YMRS items: Item 2, item 3, item 4, item 5, item 6, item 7, item 8, item 9, item 10, item 11

^*^Remained statistically significant after adjustment and Bonferroni correction for multiple testing (p < 0.005)

#### Irritability

We investigated the individual hypomanic/manic symptoms (individual YMRS items) and their relationship with irritability (see Table 5). In the univariate analysis all items except item 11 (Insight) showed an association with irritability before adjustment. After adjustment and Bonferroni correction, only item 7 (Language-Thought Disorder) remained statistically significantly associated with irritability with a p-value < 0.005. For every 1-value increasement in Language-Thought Disorder on Item 7, the odds of experiencing increased irritability was increased 1.5-fold after adjustment (adjusted OR [95% CL] 1.5 [1.3, 1.9]) (see Table 5).

**Table 5:** Odds ratios of experiencing irritability depending on the different manic symptoms (YMRS items) during 849 visits

		OR	95% confidence limits	Range of scale^a^
Item 1 (Mood)	Unadjusted	1.6	(1.4, 2.0)	0–4
	Adjusted^b^	1.1	(0.8, 1.4)	
Item 2 (Energy/activity)	Unadjusted	1.6	(1.4, 2.0)	0–4
	Adjusted	1.1	(0.8, 1.4)	
Item 3 (Sexual interest)	Unadjusted	1.6	(1.3, 2.1)	0–3
	Adjusted	1.2	(0.9, 1.6)	
Item 4 (Sleep)	Unadjusted	1.6	(1.3, 2.0)	0–4
	Adjusted	1.3	(1.0, 1.6)	
Item 5 (Irritability)	X	x	x	0–6
Item 6 (Speech)	Unadjusted	1.4	(1.2, 1.5)	0–6
	Adjusted	1.1	(0.9, 1.2)	
Item 7 (Language-Thought Disorder)	Unadjusted	1.8	(1.6, 2.2)	0–4
	Adjusted	1.5*	(1.3, 1.9)*	
Item 8 (Content)	Unadjusted	1.5	(1.3, 1.8)	0–8
	Adjusted	1.0	(0.8, 1.3)	
Item 9 (Aggression)	Unadjusted	2.5	(1.4, 4.5)	0–5
	Adjusted	2.2	(1.1, 4.2)	
Item 10 (Appearance)	Unadjusted	2.4	(1.3, 4.6)	0–4
	Adjusted	1.2	(0.6, 2.6)	
Item 11 (Insight)	Unadjusted	1.2	(0.8, 1.9)	0–6
	Adjusted	0.7	(0.4, 1.3)	

Outcome irritability > 0 vs irritability = 0

^a^Range of scale: observed minimum and maximum values of the individual YMRS items in data set

^b^Adjusted model is adjusted for all the remaining YMRS items: Item 1, Item 2, item 3, item 4, item 6, item 7, item 8, item 9, item 10, item 11

^*^Remained statistically significant after adjustment and Bonferroni correction for multiple testing (p < 0.005)

Overall, Tables 4, 5 illustrate and substantiate that elevated mood is associated with activity, which is not the case for irritability.

## Discussion

DSM-5 criterion A for hypomania/mania now includes the requirement of increased energy or activity in addition to mood change. The clinical consequences of the modification to the current DSM-5 criterion A for hypomania/mania has never been investigated in newly diagnosed bipolar patients. Elucidating the consequences of applied DSM-5 criterion A for newly diagnosed bipolar patients can help us understand the disease-trajectory of newly diagnosed bipolar patients in the light of DSM-5, and soon ICD-11 criteria.

In the present study of 373 newly diagnosed BD patients we revealed 3 overall findings. First, we found that applying DSM-5 criterion A reduced the number of patients experiencing a hypomanic/manic visit by 62% at baseline and by 50% during longitudinal follow-up, compared with DSM-IV criterion A. Second, fulfilling DSM-5 criterion A for a hypomanic/manic visit during follow-up was associated with higher scores on the YMRS and associated with increased number of contributed visits. Third, the individual association between manic symptoms available from the Young Mania Rating Scale showed a significant association between elevated mood (item 1) and energy/activity (item 2), sexual interest (item 3), decreased need for sleep (item 4), and speech (item 6). Irritability (item 5) showed a significant association with language-thought disorder (item 7).

The decrease in diagnostic prevalence (62% at baseline and by 50% during follow-up) with applied DSM-5 criterion A is clinically significant in a cohort of newly diagnosed bipolar patients. Previous studies of more chronic bipolar patients also point toward a decrease in both baseline prevalence and longitudinal diagnostic prevalence with applied DSM-5 criterion A for hypomania/mania, however the decrease in the prevalence of BD using DSM-5 compared with DSM-IV was smaller (34% (Fredskild et al. 2019) and 48% (Machado-Vieira et al. 2017)) than in our study of newly diagnosed patients with BD (62% at baseline).

More severe manic symptoms (i.e., higher YMRS score) were associated with DSM-5 diagnosis during follow-up. These results may indicate that the stricter DSM-5 criterion A captures the more severe and evident hypomanic/manic visits but cuts off 50% of previously detected visits by DSM-IV criterion A.

Number of contributed visits per patient was associated with fulfillment of DSM-5 criterion A, suggesting that patients over time, were more likely to have a hypomanic/manic visit detected according to DSM-5 criterion A.

These results are in line with findings from our previous study on more chronic bipolar patients also showing that both higher YMRS scores and number of contributed visits were associated with DSM-5 diagnosis according to criterion A (Fredskild et al. 2019). Consequently, with the DSM-5 criterion A less severe BD may be overlooked, especially early during the course of illness. This may possibly result in a longer diagnostic delay (Dagani et al. 2017; Baldessarini et al. 2007), as patients may not fulfill criteria for BD until later stages of the illness. Consequently, treatment interventions will be delayed likely with poorer outcomes (Kessing et al. 2013, 2014; Kessing 2019). Nevertheless, it should be noted that in the present study we did not prove a direct association between delay in diagnosis and fulfilling DSM-5 criterion A.

The individual association between manic symptoms available from the YMRS showed a strong association between mood and energy/activity after adjustment, however, irritability and energy/activity did not prove any significant association after adjustment. This could potentially be part of the explanation of why some of the DSM-IV diagnosed hypomanic/manic visits (elevated, expansive, or irritable mood) no longer fulfill DSM-5 criterion A (elevated, expansive, or irritable mood AND energy or activity), as irritability and energy/activity did not prove an association in our data after adjustment.

### Strengths and limitations

Our study is based on updated data from the ongoing longitudinal BIO-study, including a large cohort of well-characterized patients with newly diagnosed BD. Our cohort seems representative of newly diagnosed bipolar patients with a median age of illness onset of 18 years and a median delay in diagnosis of 5 years (Dagani et al. 2017; Baldessarini et al. 2007).

The decrease in prevalence of hypomanic and manic visits from DSM-IV to DSM-5 criterion A was larger than in previous studies, this could be explained by the present study had predominance of BD II patients (68%), and the comparable studies had a majority of BD I patients (Machado-Vieira et al. 2017; Fredskild et al. 2019). The large proportion of patients with bipolar disorder, type II in the present sample reflect that a substantial proportion of patients were reffered to the Copenhagen Affective Disorder Clinic from primary care including private psychiatrists and general practice.

Standardized, validated rating scales were used to evaluate both manic and depressive symptoms, YMRS and Hamilton 17-items, respectively, as undertaken by trained M.Ds or M.Scs in Psychology Ph.D. students. The assessment of fulfilled DSM-IV and DSM-5 criterion A were based solely on the YMRS scores on individual items, thus individual YMRS scores served as a proxy for evaluating the presence/absence of a hypomanic/manic visit, although the YMRS rating scale is not designed as a diagnostic tool but rather a rating scale measuring the overall symptom severity of individual manic symptoms. We used YMRS because this scale includes specific items on both mood, activity and irritability that are all criterion A symptoms. In this regard, it should be noted that the majority of patients included in the study did not present with a mood episode at baseline (see Table 2) and the findings were based on limited observations of patients (median visits = 2; IQR, 1–3) and a period of observation with the use of scales during approximately yearly visits of a 3 days look-back. We do not have detailed data from the Structures Clinical Interview for DSM-IV-TR Axis I Disorders (First et al. 1996) so we were not able to directly compare DSM-IV-TR and DSM-5 diagnoses.

It is evident from these and our prior data (Fredskild et al. 2019) that inclusion during a mood episode and more frequent visits and a longer follow-up period would have resulted in a higher concordance between DSM-5 and DSM-IV diagnoses of hypomania/mania and BD.

Furthermore, the present study only evaluated hypomania/mania according to criterion A and did not incorporate the time frame or the criterion B symptoms in the assessment of hypomania/mania. However, criterion A is the gate criterion to evaluate the presence/absence of a manic/hypomanic state according to DSM and thus must be fulfilled in order to continue to criterion B.

Lastly, the present study incorporated an explorative analysis of associations between DSM-5 criterion A fulfillment and clinical characteristics/demographics. Our logistic regression models included multiple testing and thus an increased risk of a random significant finding (type I error). However, this was minimized by applying Bonferroni correction for multiple testing.

On the other hand, several of our predictors showed borderline significance in the logistic regression model, which can either be a random finding, or the result of a too small sample size to detect an actual difference (type II error). Nevertheless, our findings are consistent with results from a cohort of more chronic bipolar patients published by our study group (Fredskild et al. 2019).

## Conclusions

In this prospective cohort study, applying DSM-5 criterion A for hypomania/mania reduced the diagnostic prevalence of hypomanic/manic visits by 62% at baseline and by 50% during longitudinal follow-up, compared with DSM-IV criterion A. Fulfilling DSM-5 criterion A was associated with more severe manic symptoms and increased during follow-up. Overall, fewer hypomanic/manic visits may be detected in newly diagnosed bipolar patients with applied DSM-5 criterion A, and the hypomanic/manic visits detected may be of more severe character, possibly overlooking less evident hypomanic/manic episodes. Consequently, the DSM-5 and the upcoming ICD-11, may possibly result in longer diagnostic delay of BD as compared with the DSM-IV.

## Data Availability

Data are not available.

## Abbreviations

BD: Bipolar disorder
BD I: Bipolar disorder I
BD II: Bipolar disorder II
BIO: Bipolar Illness Onset study
CADIC: Copenhagen Affective Disorder Clinic
DSM-IV: Diagnostic and Statistical Manual of Mental Disorders version IV
DSM-5: Diagnostic and Statistical Manual of Mental Disorders 5th edition
ICD-10: The International Classification of Diseases 10
ICD-11: The International Classification of Diseases 11
